# Evaluation of the specificity of a commercial ELISA for detection of antibodies against porcine respiratory and reproductive syndrome virus in individual oral fluid of pigs collected in two different ways

**DOI:** 10.1186/s12917-015-0388-7

**Published:** 2015-03-19

**Authors:** Tatjana Sattler, Eveline Wodak, Friedrich Schmoll

**Affiliations:** Large Animal Clinic for Internal Medicine, University of Leipzig, An den Tierkliniken 11, 04103 Leipzig, Germany; Institute for Veterinary Disease Control, AGES, Robert-Koch-Gasse 17, 2340 Mödling, Austria

**Keywords:** PRRSV, ELISA, Swine, Cotton gauze swabs, GenoTubes, Sensitivity

## Abstract

**Background:**

The monitoring of infectious diseases like the porcine reproductive and respiratory syndrome (PRRS) using pen-wise oral fluid samples becomes more and more established. The collection of individual oral fluid, which would be useful in the monitoring of PRRSV negative boar studs, is rather difficult. The aim of the study was to test two methods for individual oral fluid collection from pigs and to evaluate the specificity of a commercial ELISA for detection of PRRSV antibodies in these sample matrices. For this reason, 334 serum samples from PRRSV negative pigs (group 1) and 71 serum samples from PRRSV positive pigs (group 2) were tested for PRRSV antibodies with a commercial ELISA. Individual oral fluid was collected with a cotton gauze swab from 311 pigs from group 1 and 39 pigs from group 2. Furthermore, 312 oral fluid samples from group 1 and 67 oral fluid samples from group 2 were taken with a self-drying foam swab (GenoTube). The recollected oral fluid was then analysed twice with a commercial ELISA for detection of PRRSV antibodies in oral fluid.

**Results:**

All serum samples from group 1 tested negative for PRRSV antibodies. The collection of oral fluid was sufficient in all samples. Sampling with GenoTubes was less time consuming than sampling with cotton gauze swabs. False positive results were obtained in 7 (measure 1) respectively 9 (measure 2) oral fluid samples recollected from cotton gauze swabs and in 9 and 8 samples from GenoTubes. The specificity of the oral fluid ELISA was 97.4% for cotton gauze swabs and 97.3% for GenoTubes. 70 out of 71 serum samples and all oral fluid samples from group 2 tested positive for PRRSV antibodies. The sensitivity of the oral fluid ELISA was 100%. According to the kappa coefficient, the results showed an almost perfect agreement between serum and oral fluid collected in both ways (kappa > 0.8).

**Conclusions:**

Both methods used for individual oral fluid collection proved to be practical and efficient and can be used for PRRSV antibody detection. It has to be considered, however, that false positive results may occur more often than in serum samples.

## Background

In recent years, the applicability of oral fluid samples for diagnostics of infectious disease like porcine reproductive and respiratory syndrome (PRRS), caused by the PRRS virus (PRRSV) has seen increased discussion in scientific literature. Several methods of detecting PRRSV RNA and PRRSV antibodies (Ab), using both different molecular diagnostic methods and serological techniques, were developed [1-3]. Sampling techniques were evaluated [4] and the effect of the stabilization of the oral fluid [5] and sample processing [6] was determined with the intention to improve the results. Different ropes for the oral fluid collection were tested [6,7]. Some ELISAs, specifically developed for PRRSV Ab detection in oral fluid, show results comparable to serum ELISAs [2,8]. The usage of cotton ropes as chewing material for oral fluid collection was found to be the method of choice [6,7]. This system is highly suitable for pen-wise oral fluid collection in weaning pigs and fatteners. For individual oral fluid collection, however, especially from sows and boars, the animals have to be trained to chew on the cotton rope [4,7]. This is a time consuming measure and is not widely accepted among European pig producers. On the other hand, the continuous testing of individual animals via oral fluid sampling would be a substantial improvement in the monitoring in PRRSV negative herds like boar studs. This presupposes an easy, rapid, animal friendly and efficient sampling method as well as the uncomplicated storage and transport of the samples. Self-drying foam swabs like GenoTubes Livestock (Prionics, Schlieren, Switzerland) that were developed for the detection of minimal DNA amounts in forensic medicine have a small sample volume and can be stored at room temperature for several weeks [9].

For the collected oral fluid samples, test systems with a high specificity and sensitivity are needed, as they are continuously developed and improved for serum samples [10,11]. A recently developed ELISA detecting IgG Ab against PRRSV in individual oral fluid collected with cotton ropes has according to Kittawornrat et al. [8] a specificity of 100% (95% confidence interval at 99%, 100%) and a sensitivity of 94.7% (92.4%, 96.5%). According to the manufacturer of the cited IDEXX PRRS OF ELISA (IDEXX, Ludwigsburg, Germany), the specificity is quoted at 98.7% (92.2%, 100%) in 77 tested individual oral fluid samples whereas the sensitivity is 100% (94.2%, 100%) in 78 tested samples. For the IDEXX PRRS X3 Ab test (IDEXX), which is generally considered to be the de facto gold standard ELISA in the detection of PRRSV Ab in serum, the manufacturer quotes a sensitivity of 98.8% and a specificity of 99.9%.

The objective of the study was to test the efficacy and practicability of oral fluid collection from individual pigs via cotton gauze swabs and a dry foam swab (GenoTube) as well as the re-collection of oral fluid from these materials. Furthermore, the specificity of the IDEXX PRRSV OF ELISA for the detection of PRRSV Ab in oral fluid collected with these methods was evaluated in comparison to the IDEXX PRRS X3 in serum samples. To ensure the sensitivity of the oral fluid ELISA, a number of PRRSV positive pigs were tested as well.

## Methods

### Animals and serum samples

A total of 395 pigs (405 samples) were included in the study. The pigs consisted of 2 groups. Group 1 (n = 334) included 152 boars from four German boar studs, 67 boars from one Austrian boar stud, 35 fatteners from one German pig breeding farm and 57 sows and gilts as well as 23 nursery piglets from two Austrian pig breeding farms. All farms were classified as PRRSV negative (category IV according to Holtkamp et al. [12]). Group 2 included a total of 71 samples from the following pigs: a) 39 fatteners from one Austrian and one German PRRSV positive fattening farm, b) 12 nursery piglets injected with a PRRSV type 2 strain at pre-vaccine stage and c) 20 fatteners challenged with a highly pathogenic PRRSV type 2 strain. Ten of the pigs mentioned under c) were the same as in b) and used twice for sampling with a time lag of 28 days between both sampling times. A blood sample was taken from each pig. All blood samples, except of the pigs mentioned under b) and c) in group 2, were collected in the course of monitoring programs and not taken for the purpose of this study. Housing, animal care and experimental protocol of the pigs mentioned under b) and c) were approved by the local ethics committee (Agency of the Government in Lower Austria, Department of Agrarian Law). Blood samples were centrifuged for 10 minutes at 2400 g within 4 hours after sampling and serum was kept frozen at minus 20°C until analysis.

### Collection and handling of oral fluid samples

**Figure 1:**
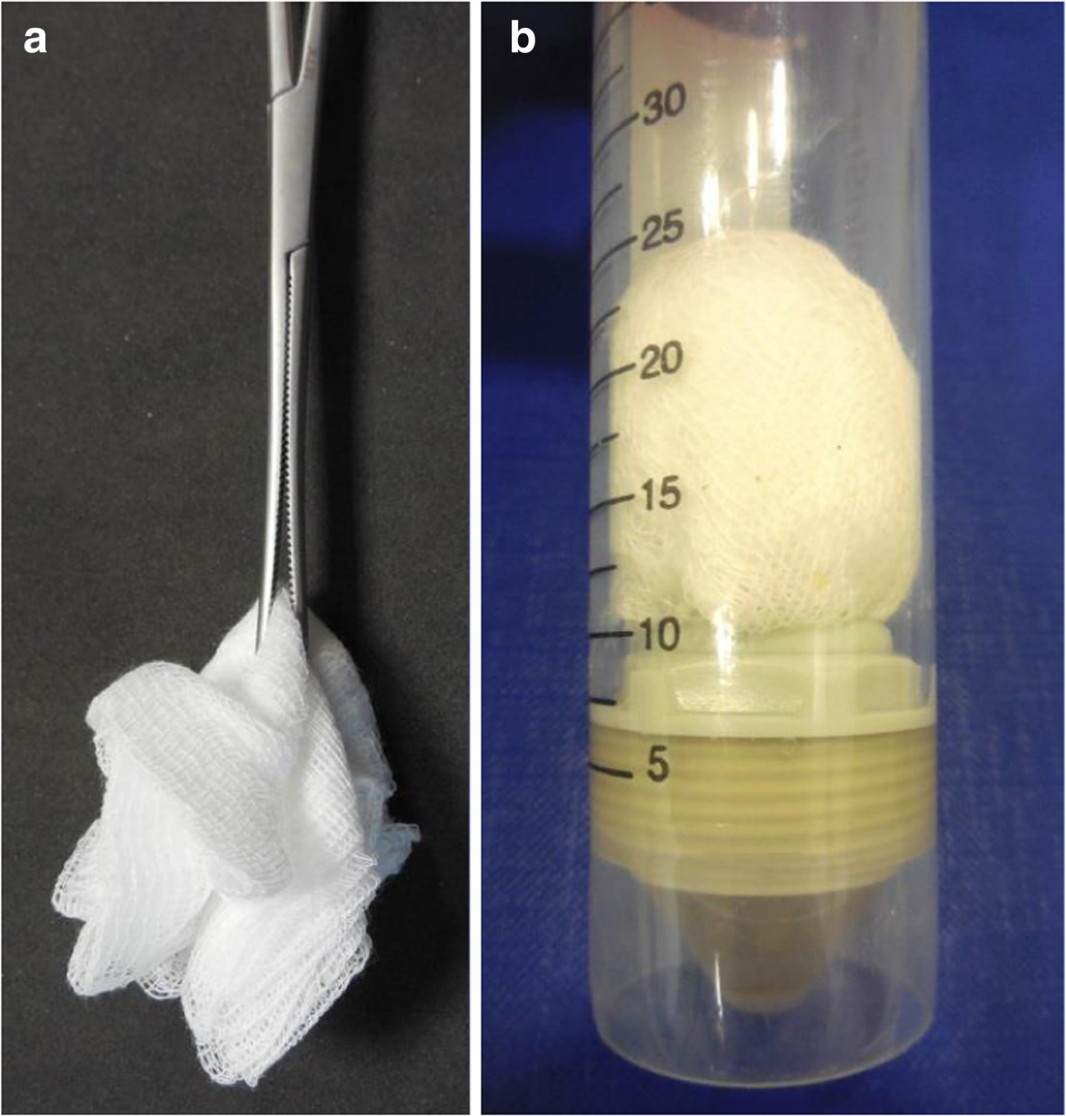
**Oral fluid collection via cotton gauze swabs (a), centrifugation for re-collection of oral fluid (b).**

**Figure 2:**
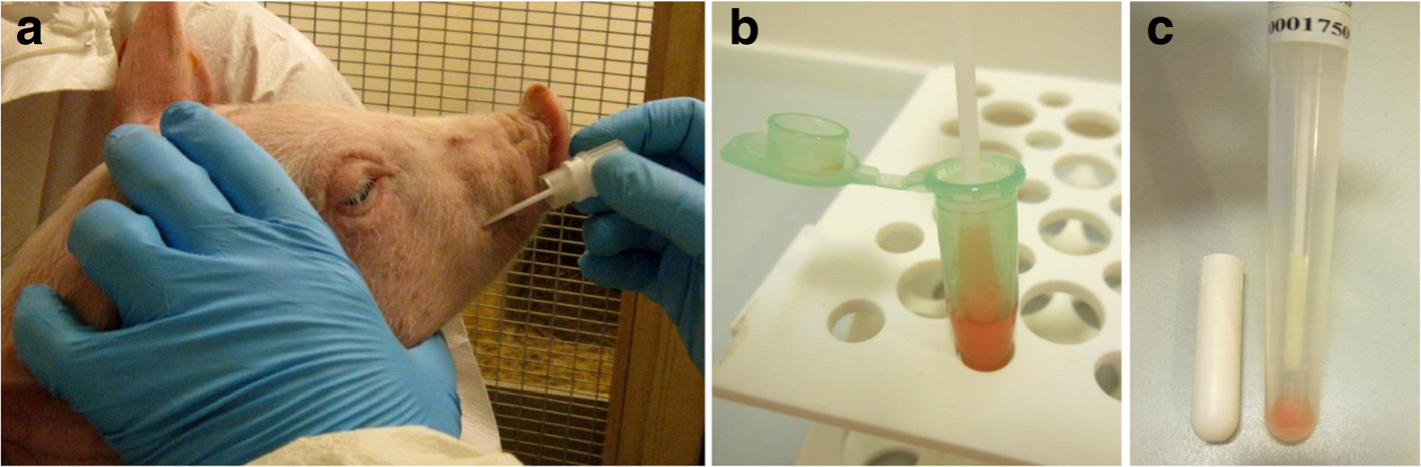
**Oral fluid collection via GenoTubes (a), reconstitution of oral fluid by resuspension (b) and following centrifugation (c).**

Oral fluid was collected from the above mentioned pigs in two different ways while they were fixated for blood sampling or, in case of the boars, during semen collection:

1. Individual oral fluid samples were collected via cotton gauze swab. For this purpose, the swab was held into the mouth of the respective pig with a serrefine and the oral fluid was allowed to soak into the swab (Figure 1a). The swabs were stored in a 50-ml-falcon tube at minus 20°C until re-collection of the oral fluid and analysis. For the re-collection of the oral fluid, the swab was centrifuged for 10 minutes at 2500 g in a 50-ml-falcon tube with filter (Figure 1b).

2. Individual oral fluid samples were collected via GenoTubes (Figure 2a). The GenoTubes soaked with oral fluid were stored at room temperature up to four weeks until analysis.

Group 1: In 289 pigs oral fluid could be collected in both described ways. In 22 pigs (boars from Austria) only cotton gauze swabs were used and in another 23 pigs (nursery piglets from Austria) only GenoTubes were utilised. Group 2: Oral fluid samples were collected from 35 pigs both via cotton gauze swab and via GenoTube. In another 4 pigs only cotton gauze swabs and in 22 pigs (32 samples) only GenoTubes were collected.

Oral fluid collection was done on the same day that the blood samples were taken from the respective pigs.

### Detection of PRRSV antibodies by ELISA

All serum samples were analysed with the IDEXX PRRS X3 Ab test for the presence of antibodies against PRRSV.

All oral fluid samples were analysed with the IDEXX PRRS OF ELISA, designed for detection of antibodies against PRRRSV in oral fluid. To test the reproducibility of results, samples of group 1 were tested in two different measures. The capacity of the foam swab of the GenoTube was measured experimentally. For this reason, 10 GenoTubes were dived into oral fluid for some seconds and the amount of fluid soaked into the swab was measured with weighing. The average was at approximate 200 μl with no considerable deviation. To reconstitute the dried oral fluid, the foam swab of the GenoTube was re-suspended in a 1.5 ml microcentrifuge tube with 400 μl of the dilution buffer of the ELISA kit (Figure 2b) which means a 1:2 dilution of the contained oral fluid as is required in manufacturer’s instructions. To remove the remaining oral fluid from the foam swab, the GenoTubes were centrifuged for 10 minutes at 2500 g after removing the SafeDry medium from the tube (Figure 2c). The gained fluid was added into the respective microcentrifuge tube.

All serum and oral fluid ELISAs were conducted according to the manufacturer’s instructions. A brief description of the IDEXX PRRSV OF ELISA is given in [1]. In both ELISAs, samples with sample-to-positive (S/P) ratios ≥0.4 (cut-off value) were considered positive for PRRSV antibodies.

### Statistical analysis

The specificity of the IDEXX PRRS OF ELISA in oral fluid from cotton gauze swabs and GenoTubes compared to the IDEXX PRRS X3 Ab test in serum was estimated using group 1. The sensitivity of the IDEXX PRRS OF ELISA in oral fluid from cotton gauze swabs and GenoTubes compared to the IDEXX PRRS X3 Ab test in serum was tested using the samples from group 2. The correlation of S/P values of the ELISAs were tested in group 2 with the correlation coefficient after Spearman. Over all samples, the accuracy of the IDEXX PRRS OF ELISA in oral fluid from cotton gauze swabs and GenoTubes was calculated. In measure one, the agreement of the IDEXX PRRS OF ELISA in oral fluid from cotton gauze swabs and GenoTubes with the IDEXX PRRS X3 Ab test in serum was determined with the kappa coefficient (κ) and interpreted according to Landis and Koch [13].

## Results

### Collection of oral fluid samples

From all cotton gauze swabs, a sufficient amount of oral fluid (between 0.5 and 5.0 ml) could be collected. The collection from each pig took between 30 seconds and three minutes. The limiting factor for the collection of oral fluid samples via cotton gauze swabs was the dryness of the mouth of the respective pig. This was more often the case in smaller pigs while the mouths of sows and especially breeding boars contained more oral fluid. The collection of oral fluid via cotton gauze swabs from boars during semen collection is possible without any fixation.

The collection of oral fluid with GenoTubes took only a few seconds and went therefore much faster than with cotton gauze swabs. The usage of GenoTubes in fatteners and adult pigs is mostly possible without fixation of the pig.

### Detection of PRRSV antibodies by ELISA

The serum samples of all group 1 pigs tested negative for PRRSV antibodies. The calculated specificity of the serum ELISA was therefore 100%.

The results of the oral fluid ELISA from cotton gauze swabs and GenoTubes compared to the serum ELISA are shown in Table 1. The S/P values in the PRRSV antibody negative oral fluid samples ranged from 0.00 to 0.39 both in cotton gauze swabs and in GenoTubes. S/P values from false positive oral fluid samples from cotton gauze swabs ranged from 0.40 to 0.95, those from GenoTubes ranged from 0.41 to 0.84. In cotton gauze swabs as well as in GenoTubes, respectively, five false positive samples agreed between measure 1 and 2. No agreement of false positive samples was found between cotton gauze swabs and GenoTubes.

**Table 1:** **Two-by-two contingency table comparing results of ELISA for detection of PRRSV antibodies in serum and oral fluid collected via cotton gauze swabs and GenoTubes**

**IDEXX PRRS OF - oral fluid**			**IDEXX PRRS X3 Ab - Serum**	
			**Negative**	**Positive**
**Cotton gauze swabs**	Measure 1	Negative	304	0
		Positive	7	39
	Measure 2	Negative	302	-
		Positive	9	-
		Total	311	39
**GenoTubes**	Measure 1	Negative	303	0
		Positive	9	67
	Measure 2	Negative	304	-
		Positive	8	-
		Total	312	67

**Figure 3:**
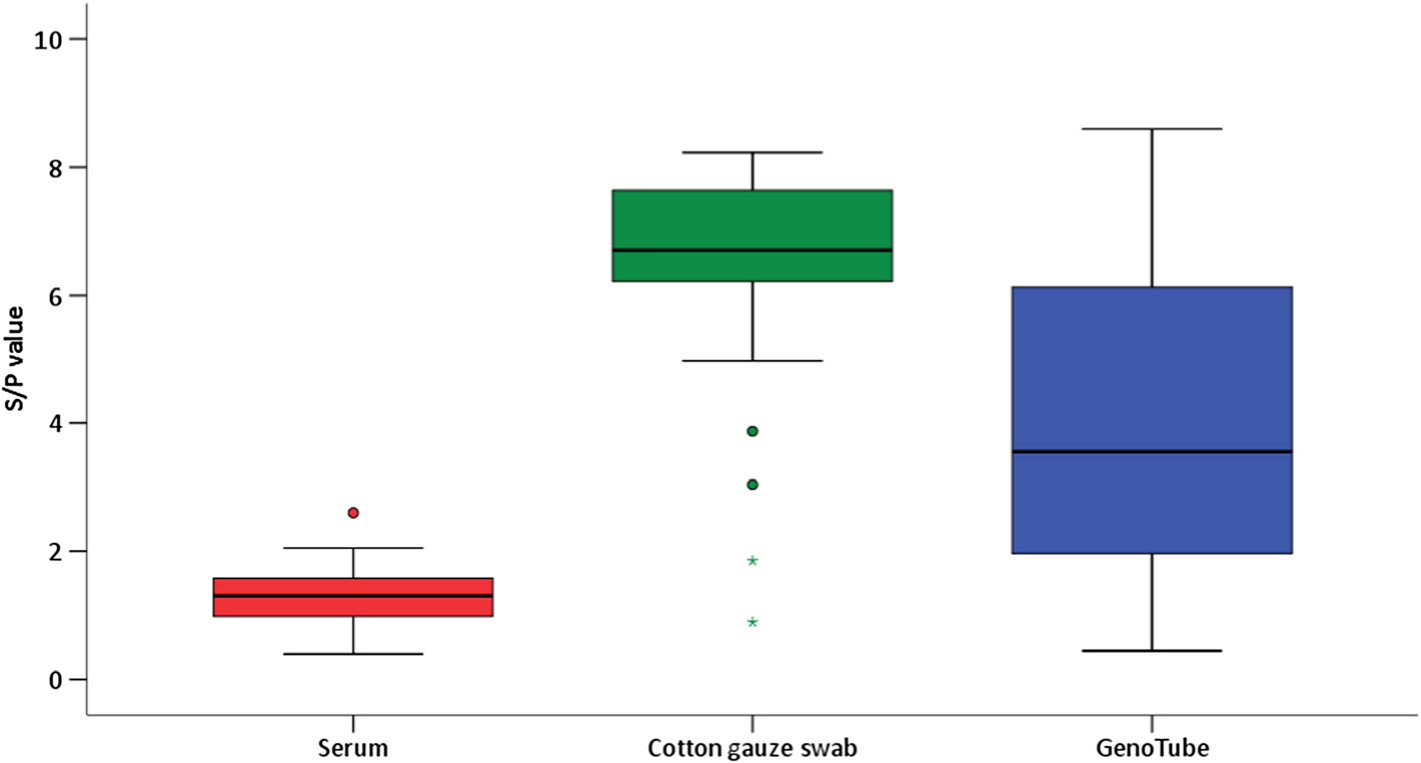
**PRRSV antibodies in serum and oral fluid collected via cotton gauze swab and GenoTubes in PRRSV positive pigs.**

The S/P values of the PRRSV antibody ELISAs in group 2 can be seen in Figure 3. 70 of the 71 serum samples, all cotton gauze samples and all GenoTubes were tested positive for PRRSV antibodies. The S/P values of the positive samples in serum ranged from 0.48 to 2.60, in cotton gauze swabs from 0.89 to 8.93 and in GenoTubes from 0.44 to 8.60. The negative serum sample had a S/P value of 0.39, the S/P value of the corresponding Genotube was at 3.94. There was a positive correlation of S/P values between serum and GenoTubes (r = 0.40) and between cotton gauze swabs and GenoTubes (r = 0.82) in the samples of group 2.

Descriptive test parameters and measurements of agreement for all three samplings are shown in Table 2.

**Table 2:** **Descriptive test parameters and measures of agreement of the IDEXX PRRSV OF using oral fluid collected via cotton gauze swabs and GenoTubes**

	**Cotton gauze swabs**	**GenoTubes**
Specificity (%)	97.4	97.3
Sensitivity (%)	100.0	100.0
Accuracy (%)	97.7	97.6
Kappa coefficient (κ)	0.91	0.91

IDEXX PRRS X3 Ab in serum was used as reference test.

## Discussion

In this study, two different ways of individual oral fluid sampling were evaluated for their effectiveness and practicability. Using these oral fluid samples, the descriptive test parameters of the IDEXX PRRSV OF ELISA for detection of PRRSV antibodies in oral fluid were calculated in comparison to the IDEXX PRRSV X3 ELISA in serum samples.

Until now, besides the usage of GenoTubes Livestock for DNA analysis of the tested individuals, only a few studies described the use of GenoTubes for detection of infectious agents in animals [14,15]. In the mentioned studies, *Brachyspira* DNA in rectal swabs of pigs, respectively classical and African swine fever virus DNA in wild boars were detected by PCR. One study is published that describes the usage of GenoTubes for sampling and detection of antibodies against African swine fever virus [16]. However, no study referring to the usage of GenoTubes for PRRSV antibody detection by ELISA was available until now.

Both of the sampling techniques used, cotton gauze swabs as well as GenoTubes, proved to be efficient for oral fluid collection from individual pigs. The collection by both sampling methods was successful in all pigs. Individual oral fluid collection by cotton or polyester ropes is not always that successful even in trained pigs (success between 37.5 and 87.5% of the cases) [17]. The collection via cotton gauze swabs, however, was time consuming and more difficult in smaller pigs than in fatteners or adult pigs. Sampling adult boars without fixation is possible for instance during semen collection. It has to be considered, however, that the swab must be taken from within the mouth. Collection of frothy saliva around the mouth was proven to be insufficient in other studies [4]. The collection of oral fluid with GenoTubes was easier and less time consuming than with cotton gauze swabs and can be done in larger and adult pigs mostly without the fixation of the animal. The re-collection of oral fluid from GenoTubes was efficient and can be standardised. The GenoTube contains a SafeDry medium that causes a rapid active drying of the sample. The absence of fluid makes the samples very stable. Samples collected with a GenoTube can therefore be stored for several weeks and transported at room temperature [9].

According to the kappa coefficient, almost perfect agreement (κ > 0.80) [13] was found between ELISA results in serum and oral fluid from cotton gauze swabs and GenoTubes. The sensitivity of the ELISA was 100% in both oral fluid sample species. This number agrees with the sensitivity given by the manufacturer of the ELISA for oral fluid collected with cotton ropes. The one serum sample of group 2 that was PRRSV Ab negative in serum had a S/P value slightly beneath the cut-off, whereas the corresponding GenoTube sample was found clearly positive. It has to be considered, however, that for an accurate analysis of sensitivity a larger number of samples must be analysed. Other studies defined the sensitivity of the ELISA with 94.7%, tested in PRRSV type 2 antibody positive samples exclusively [8], and with 94.7% (n = 19) in cotton ropes and 93.3% (n = 15) in polyester ropes, tested in PRRSV type 1 inoculated pigs [17], and were thereby lower than calculated in this study. Some false positive results can occur by analysing PRRSV antibodies in oral fluid by ELISA. The specificity of the ELISA in this study is with 97.4% for cotton gauze swabs and 97.3% for GenoTubes within the confidence interval given by the manufacturer but lower than calculated in other studies for oral fluid samples collected with cotton ropes [8]. In group 2, a correlation was found between the S/P values of serum samples and oral fluid collected with GenoTubes. This underlines the good agreement between serum and oral fluid samples found in other studies as well [17,18]. There was a strong correlation between S/P values of PRRSV ab positive oral fluid samples collected with GenoTubes and with cotton gauze swabs, confirming the reproducibility of the results of the oral fluid ELISA in samples collected with different sampling methods. The sampling techniques used in this study are therefore equally suitable for oral fluid collection and subsequent testing with the IDEXX PRRS OF ELISA as cotton ropes.

## Conclusions

This study shows that oral fluid samples can be used for the PRRSV antibody detection on individual pig level. The use of GenoTubes proved to be an especially practical method both for oral fluid collection and for storage and transport of the samples. The ELISA detecting PRRSV Ab in oral fluid collected by cotton gauze swabs and GenoTubes proved to be highly sensitive. It has to be considered, however, that false positive samples needing re-testing may occur. This can cause irritations, especially in PRRSV negative farms like boar studs.
